# Eosinophils Are Important for Protection, Immunoregulation and Pathology during Infection with Nematode Microfilariae

**DOI:** 10.1371/journal.ppat.1003988

**Published:** 2014-03-13

**Authors:** Emma T. Cadman, Katherine A. Thysse, Siobhan Bearder, Anita Y. N. Cheung, Ashleigh C. Johnston, James J. Lee, Rachel A. Lawrence

**Affiliations:** 1 The Royal Veterinary College, Department of Comparative Biomedical Sciences, London, United Kingdom; 2 Division of Pulmonary Medicine, Department of Biochemistry and Molecular Biology, Mayo Clinic Arizona, Scottsdale, Arizona, United States of America; National Institutes of Health, United States of America

## Abstract

Eosinophil responses typify both allergic and parasitic helminth disease. In helminthic disease, the role of eosinophils can be both protective in immune responses and destructive in pathological responses. To investigate whether eosinophils are involved in both protection and pathology during filarial nematode infection, we explored the role of eosinophils and their granule proteins, eosinophil peroxidase (EPO) and major basic protein-1 (MBP-1), during infection with *Brugia malayi* microfilariae. Using eosinophil-deficient mice (PHIL), we further clarify the role of eosinophils in clearance of microfilariae during primary, but not challenge infection *in vivo*. Deletion of EPO or MBP-1 alone was insufficient to abrogate parasite clearance suggesting that either these molecules are redundant or eosinophils act indirectly in parasite clearance via augmentation of other protective responses. Absence of eosinophils increased mast cell recruitment, but not other cell types, into the broncho-alveolar lavage fluid during challenge infection. In addition absence of eosinophils or EPO alone, augmented parasite-induced IgE responses, as measured by ELISA, demonstrating that eosinophils are involved in regulation of IgE. Whole body plethysmography indicated that nematode-induced changes in airway physiology were reduced in challenge infection in the absence of eosinophils and also during primary infection in the absence of EPO alone. However lack of eosinophils or MBP-1 actually increased goblet cell mucus production. We did not find any major differences in cytokine responses in the absence of eosinophils, EPO or MBP-1. These results reveal that eosinophils actively participate in regulation of IgE and goblet cell mucus production via granule secretion during nematode-induced pathology and highlight their importance both as effector cells, as damage-inducing cells and as supervisory cells that shape both innate and adaptive immunity.

## Author Summary

Eosinophil recruitment is a classic characteristic of both allergic and parasitic helminth diseases. Elucidation of the role of eosinophils in these diseases is of pivotal importance for understanding the mechanisms of protection and the development of pathology. In the last few years, the part played by eosinophils in helminth-defence has been dissected using *in vivo* models and their importance in protection has been shown to be highly specific to the host-parasite combination. This study dissects the role of eosinophils during infection with the human lymphatic filarial parasite, *Brugia malayi*, which causes the major neglected tropical disease, lymphatic filariasis. In particular, we study the role of the eosinophil as a double–edged sword in generating both protection and pathology. We definitively confirm the importance of eosinophils in protection against *B. malayi* microfilariae and show that protection is not mediated by release of the eosinophil granule proteins, major basic protein or eosinophil peroxidase alone. Overall, we reveal that during an infection with *B. malayi* microfilariae, eosinophils are critical for primary protective responses. However, eosinophils contribute to nematode-induced lung dysfunction, while additionally, eosinophil granules are important negative regulators of parasite-induced lung inflammatory and some adaptive immune responses.

## Introduction

Eosinophilia is one of the principal features of parasitic helminth infection and is also associated with asthmatic disease and many gastro-intestinal disorders. However, for many years the relative role of eosinophils in protection against parasites and/or pathology has been contentious. Eosinophils are integral to the development of asthmatic pathology [1], [2], yet are commonly thought to be beneficial for protection against helminth infections [3], [4]. It is now known that the role of eosinophils in protective immune responses differs depending upon the species of infecting helminth [3]. We and others, have previously shown that eosinophils are required for killing of the filarial nematode, *Brugia sp.*, during primary, but not challenge infection [5], [6]. In related filarial infections, eosinophils are associated with clearance of parasites in challenge, but not primary infection [7], [8]. Eosinophils also play a role in clearance of primary *S. stercoralis* infections [9], [10], and secondary *N. brasiliensis* and *T. spiralis* infections [11], [12].

The protective function of eosinophils, has been hypothesised to be mediated by deposition of eosinophil granule contents upon the helminth surface. Indeed each of the eosinophil granule proteins, major basic protein-1 (MBP-1), eosinophil peroxidase (EPO), eosinophil-derived neurotoxin (EDN) and eosinophil cationic protein (ECP), can kill *Brugia sp.* microfilariae (Mf) *in vitro* [13]. During the eosinophil-dependent protective response in primary *in vivo* infection with either *B. malayi* Mf or *S. stercoralis* L3, EPO is released into the bloodstream [5], [9] and deficiency of EPO or MBP-1 can enhance the establishment of *L. sigmodontis* L3 in non-permissive mouse strains [14].

Despite their role in protection, eosinophils can also have detrimental effects on the host during helminth infection, either by prolonging parasite survival, increasing reproductive maturation, or by contributing to host pathology [15], [16], [17]. Development of airway hyper-responsiveness (AHR) in *B. malayi* infected mice has been shown to be dependent on IL-5 [17]. Likewise, AHR in *Toxocara canis* and *Ascaris suum* infections is associated with increased lung eosinophilia, IL-5 and IgE in mice [18], [19]. Eosinophil granule proteins may be linked with the pulmonary pathologies in these models as they can induce the production of airway remodelling factors by bronchial epithelial cells [20] and during challenge infection with *B. malayi* Mf in mice, deposition of the eosinophil granule protein, MBP-1 was observed [17].

This study addresses the role of eosinophils in nematode infection. We dissect the role of the eosinophil as a double-edged sword in generating protection and in mediating pathology. We definitively confirm the importance of eosinophils in protection against *B. malayi* Mf by using eosinophil-deficient mice (PHIL). Furthermore we investigate whether the eosinophil granule proteins, EPO and MBP-1 are necessary for protection against Mf. By using eosinophil-deficient and eosinophil granule deficient mice we revealed that eosinophils are important for protective immune responses, and equally, eosinophil granules were shown to be negative regulators of parasite-induced lung inflammatory and adaptive responses.

## Results

### Eosinophil presence is necessary for Mf clearance in primary, but not challenge, infection

**Figure 1:**
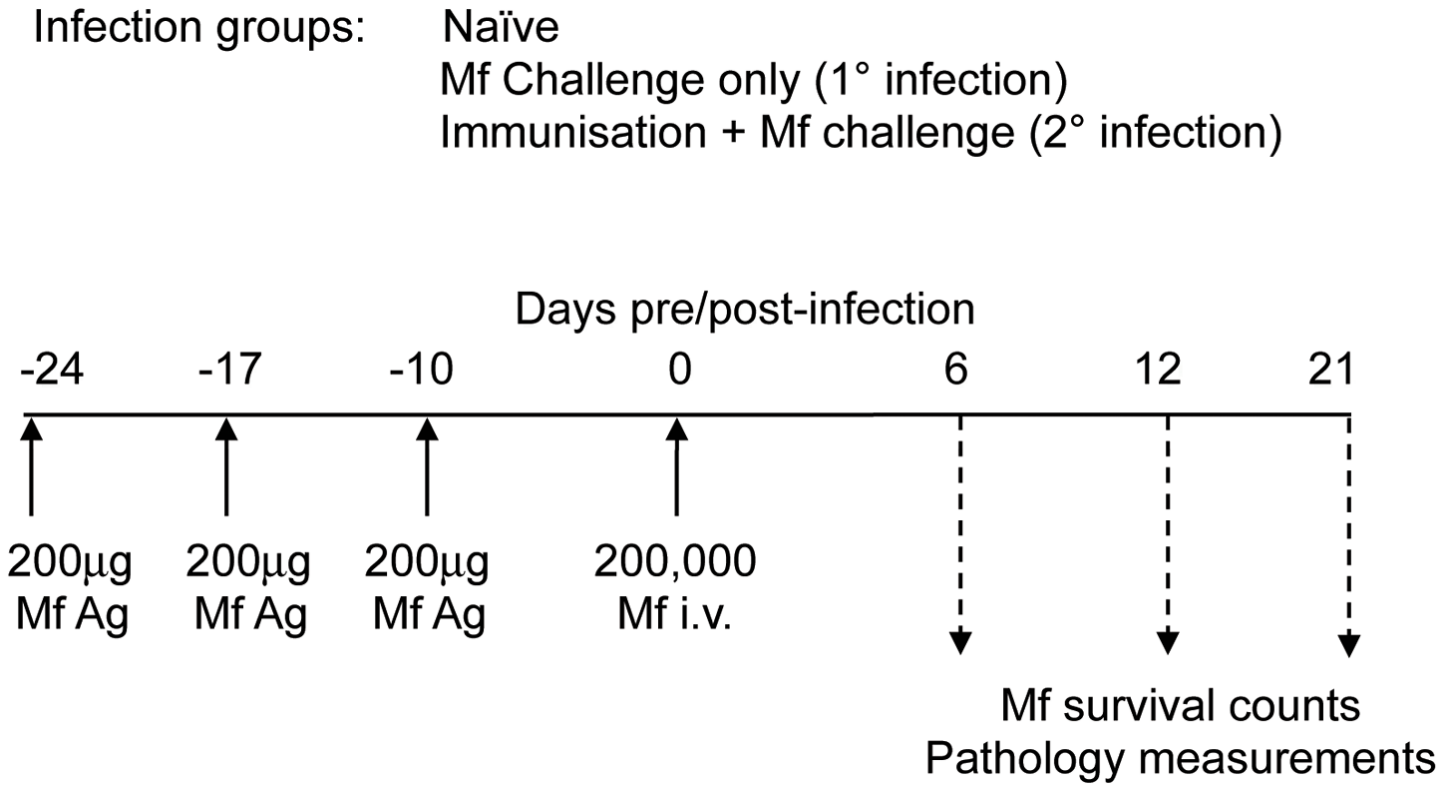
Mouse infection regimen. In each experiment, groups of four to six male C57Bl/6 wild-type mice and/or gene-targeted mice were either left uninfected (naïve), or they were injected with 200,000 *B. malayi* Mf i.v. (primary (1°) infection) or they were immunised on three occasions with 200 µg soluble Mf extract prior to challenge with 200,000 *B. malayi* Mf i.v. (challenge (2°) infection). At days 5–6, 10–12 and 20–21 post infection with Mf, immunological and pathological parameters of mice were measured. This figure shows the exact regimen used in the PHIL mice experiments.

Groups of eosinophil-less (PHIL) mice and wild-type (WT) mice were either a) left naïve and uninfected or b) given a primary live challenge infection of 200K Mf i.v., or c) they were immunised with 3×200 µg doses of MfAg and challenged i.v. with 200K live Mf (Figure 1). Mice were killed at days 6, 12, and 21 post infection and Mf were counted at each time point. Previous immunohistochemical studies, of all tissues in the mouse that have resident eosinophil populations at baseline, revealed no MBP^+^ cells in PHIL mice [1]. In addition, a sensitive EPO-based ELISA assay revealed no evidence of EPO expression in PHIL mice [21].

**Figure 2:**
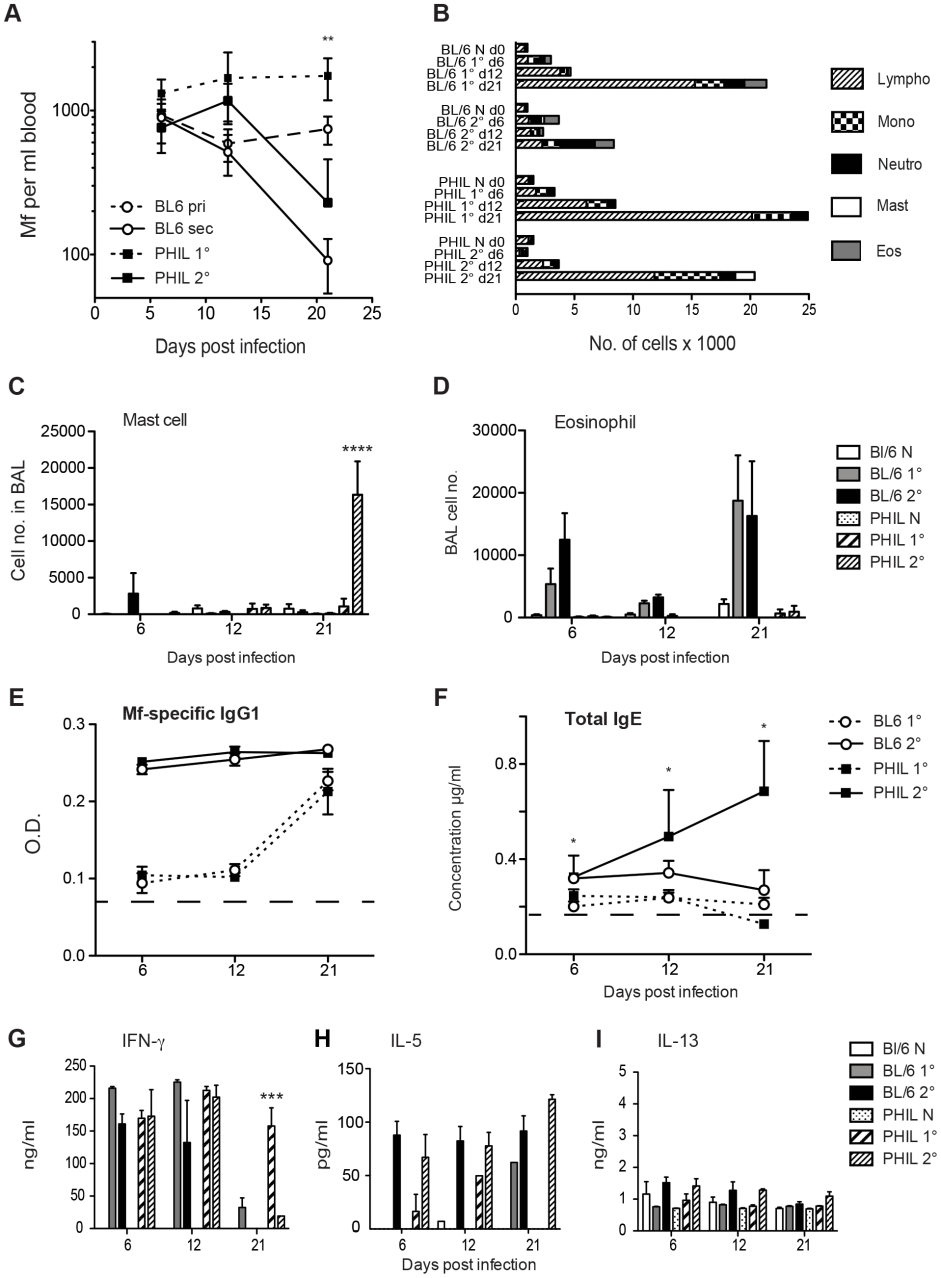
Eosinophils are required for clearance of *B. malayi* Mf during primary (1°), but not challenge (2°) infection. **A.** Mf survival in groups of PHIL (black squares) and C57Bl/6 (open circles) mice, following primary (dotted line) and secondary (solid line) infection. Mf were counted in blood from the tail vein of mice on days 6 and 12 p.i. or in cardiac blood on day 21 p.i. (d21 PHIL mice 1° versus C57Bl/6 1° p = 0.0018) **B–D.** Cell recruitment into BALF of naïve PHIL and C57Bl/6 mice and both mouse strains mice given primary or challenge Mf infections on day 21 p.i. **B.** Mean total and differential cell recruitment into BALF. **C.** Numbers of mast cells recruited into BALF (Mean ± S.E) of naïve and infected PHIL and C57Bl/6 mice. **D.** Numbers of eosinophils recruited into BALF (Mean ± S.E) of naïve and infected PHIL and C57Bl/6 mice. **E.** Mf-specific IgG1 antibody in serum during infection of PHIL and C57Bl/6 mice (Mean ± S.E.) (dotted line represents naïve levels in both PHIL and C57Bl/6). **F.** Total IgE antibody in serum during infection of PHIL and C57Bl/6 mice (Mean ± S.E.). **G–I.** Cytokines produced by splenocytes upon stimulation with Mf antigen and measured in cell culture supernatants by ELISA 72 h later. Graphs show mean ± S.E. cytokine concentration of naïve PHIL and C57Bl/6 mice and mice 21 days post primary (1°) and challenge (2°) infections of live Mf. **G.** IFN-γ responses **H.** IL-5 responses. **I.** IL-13 responses. This figure represents data from two independent experiments with 6 individual mice per group. *represents a significant difference at p<0.05, ** p<0.01 ****p<0.0001 between groups of PHIL mice and C57Bl/6 mice given the same infection regimen.

The absence of eosinophils led to significantly longer Mf survival during primary infection (P<0.01), further clarifying our previous work in IL-5 and eotaxin-1 deficient mice [5] (Figure 2A). In accord with our previous work [5], these experiments also showed that eosinophils were not essential for rapid clearance of challenge Mf infections following repeated immunisations (Figure 2A).

### Absence of eosinophils alters the phenotype of cells recruited into the broncho-alveolar lavage fluid

Blood-borne microfilariae sequester primarily in the small vessels in the lungs of their host and in human filarial patients this can lead to a severe asthmatic syndrome known as Tropical Pulmonary Eosinophilia (TPE). It has previously been demonstrated in mouse models, that Mf infections of *Brugia* sp. i.v., leads to AHR, pulmonary inflammation and a cellular infiltrate into the broncho-alveolar lumen [5], [22]. The type of cells recruited into the broncho-alveolar lavage fluid (BALF) was investigated in the naïve and infected PHIL mice. Eosinophils were recruited into the BALF of WT mice during the course of both primary and challenge infections of *B. malayi* Mf. Maximal eosinophil counts were obtained in BALF at day 21 post infection. Interestingly, in the absence of eosinophils there were no significant differences in the recruitment of other leucocyte cell types into the lung of either naïve mice or mice with primary infections. However, following immunisation plus challenge, there was a significant rise in mast cells in the BALF of mice at day 21 (P<0.0001) (Figure 2B–D). Mast cells were infrequently seen in the lungs of WT *Brugia* Mf-infected mice.

### Absence of eosinophils alters filarial-nematode induced immune responses

Antibody responses to primary Mf infection in WT mice were characterised by IgM and each of the IgG isotypes as we have shown previously [23]. Mice lacking eosinophils had similar Mf-specific antibody responses during primary infection (data not shown). Immunisation and Mf challenge resulted primarily in Mf-specific IgG1 responses in WT mice (Figure 2E). However, mice lacking eosinophils, had both strong Mf-specific IgG1 responses, and high levels of IgE, which increased over 21 days post-challenge (p<0.05) (Figure 2F). This suggests that eosinophils may play a role in downregulation of IgE during helminth infection.

When the recall response of splenocytes to Mf extract was investigated, IFN-γ was stimulated significantly in both primary and challenge infection regardless of the presence of eosinophils. This was in accord with our earlier observations [23] (Figure 2G). However in the absence of eosinophils, the IFN-γ response was sustained until day 21 during primary infection, possibly due to stimulation by prolonged parasite survival (p<0.001) (Figure 2G). Post-challenge, both WT and eosinophil-less mice had high levels of IL-5 (Figure 2H) while levels of IL-13 were very low (Figure 2I). Secretion of IL-10 by splenocytes from WT or PHIL mice during primary and challenge infection was very low and IL-4 was not detected in these experiments (data not shown).

### Absence of eosinophils alters filarial-nematode associated pathology

**Figure 3:**
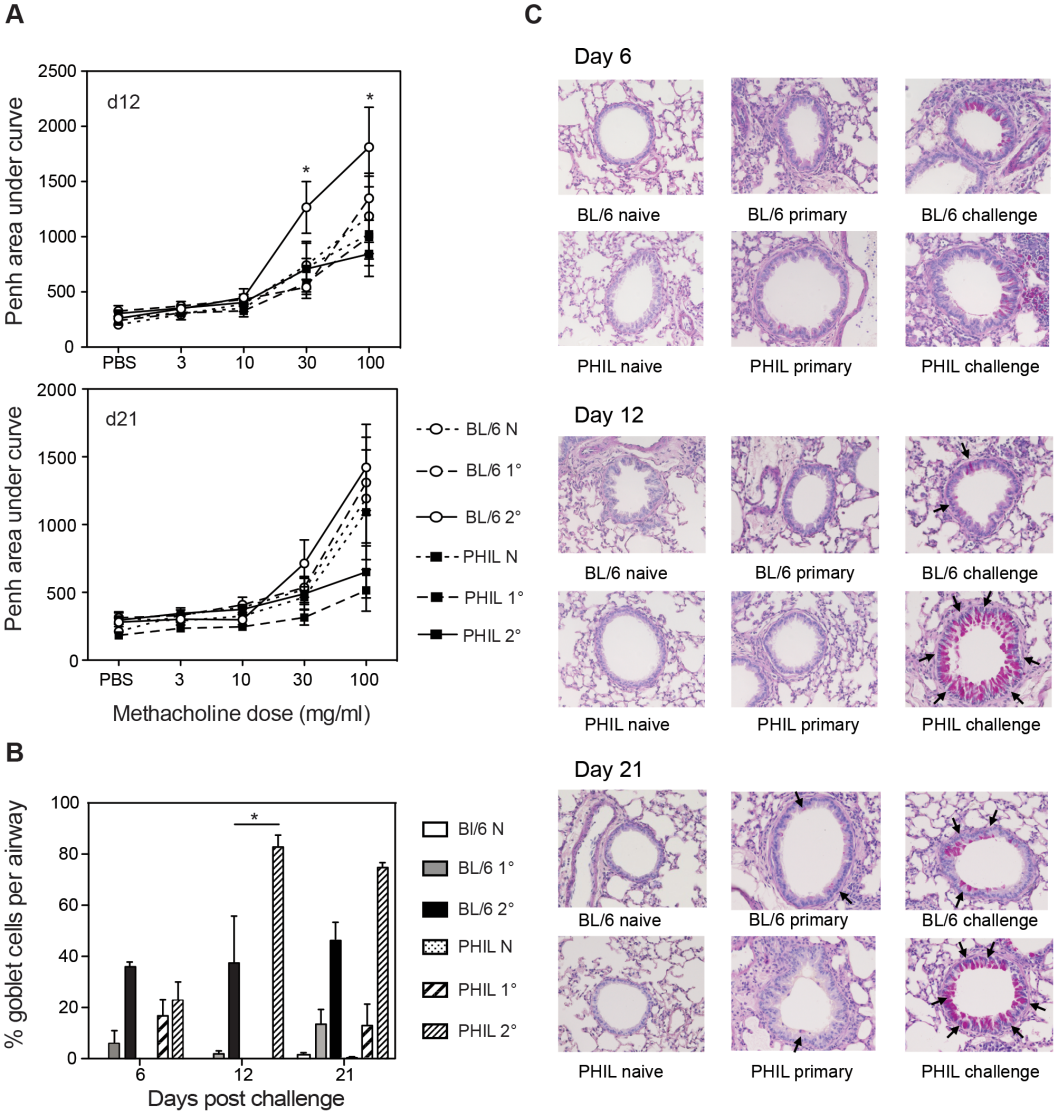
Lung function and levels of mucus-secreting goblet cells in eosinophil-less mice given *B. malayi* Mf infection. **A.** Penh values were measured in conscious, unrestrained mice administered with increasing doses of the aerosolized bronchoconstrictor, methacholine. Mean Penh ± S.E. is shown during Mf infection. **B–C.** Lungs were embedded in wax, sections were cut at 6 µm and stained with periodic acid Schiff (magnification ×40). **B.** Mean ± S.E. percentage of positively staining goblet cells per airway are shown for each infection group. **C.** The arrows show positive PAS staining at day 6, 12 and 21 post infection. This figure represents data from two independent experiments with 6 individual mice per group. *represents a significant difference at p<0.05, ** p<0.01 ****p<0.0001 between groups of PHIL mice and C57Bl/6 mice given the same infection regimen.

Filarial-nematode induced airway dysfunction was examined by whole body plethysmography at day 6, 12 and 21 post challenge. At d12, but not day 21, post-challenge, WT mice had significantly greater airway responsiveness to metacholine challenge than their eosinophil-less counterparts (P<0.05) (Figure 3A). However there were no significant differences in the plethysmography results of mice that were given live primary Mf challenge alone. Interestingly, the airway responses of all PHIL mice, and particularly the infected PHIL mice, were reduced compared to those of WT mice at day 21 post Mf-challenge, albeit these differences were not significant.

Histological sections of lungs from eosinophil-less mice were examined to determine whether there were any other histological differences in the lungs of these mice relative to WT animals. Lungs from groups of naïve, primary and challenge infected WT and PHIL mice were examined for goblet cell hyperplasia, cellular infiltration and collagen remodelling. Interestingly, there were more goblet cells in PHIL mice at day 12 and day 21 post-challenge infection and this was significant at day 12 (p<0.05) (Figure 3 B–C). This suggests that eosinophils play a part in regulating goblet cell hyperplasia. In addition, by implication goblet cells appear not to be significantly involved in the mechanical changes in lung responses because higher numbers of goblet cells in PHIL mice are associated with lower responsiveness. Collagen remodelling in primary and challenge infections in WT mice did not appear to be significantly different in comparison to eosinophil-less mice (Figure S1). Both strains of mice showed similarly increased levels of cellular infiltration and deposition of extracellular matrix proteins following infection at each time point examined. Overall these results suggest that eosinophils do play a targeted role in nematode-induced lung pathology.

### Eosinophil peroxidase is involved in respiratory physiological changes but not Mf clearance in primary infection

**Figure 4:**
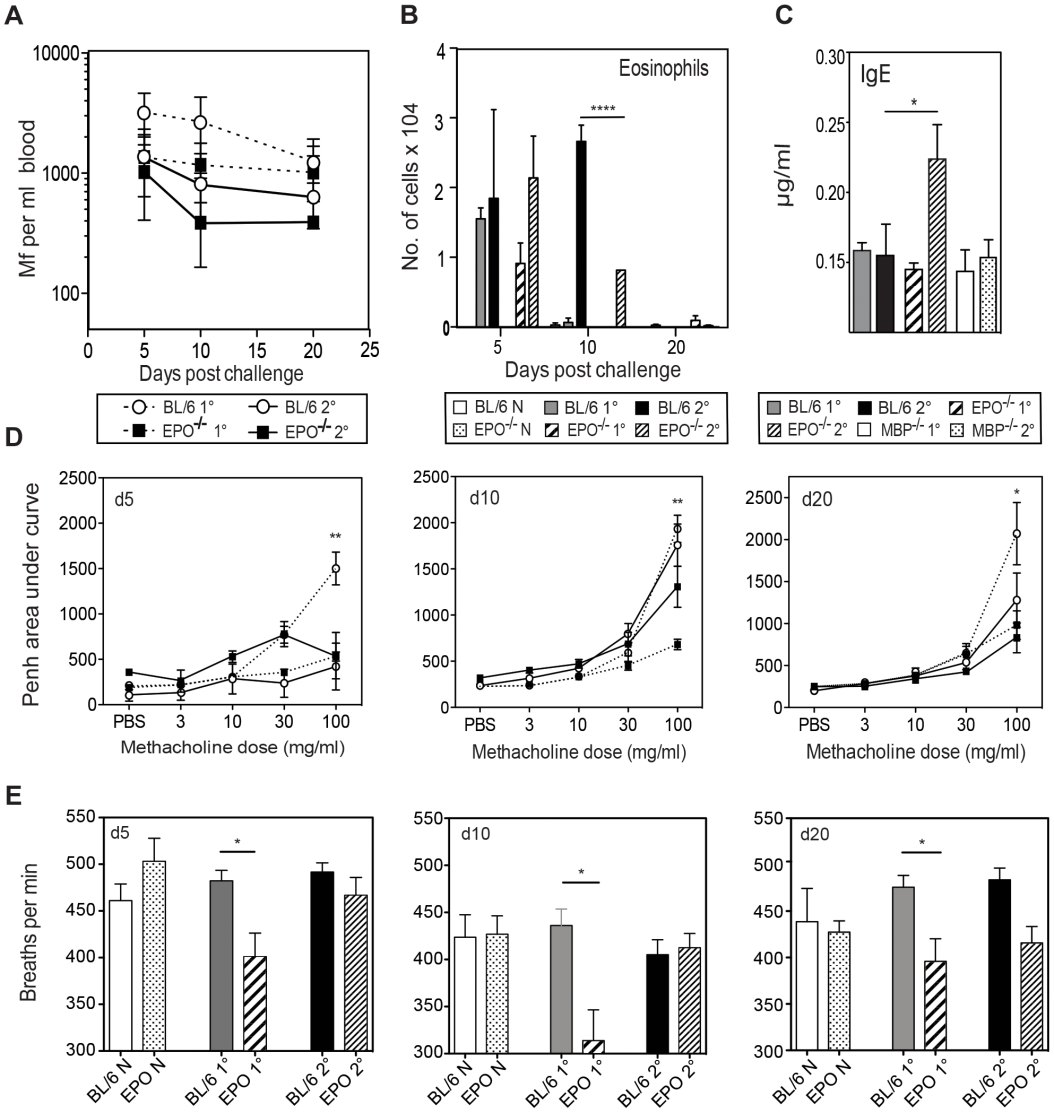
EPO is not required for Mf survival but contributes to pulmonary eosinophil recruitment, total IgE production and nematode-induced in lung physiology following Mf infection. **A.** Mf survival in EPO^−/−^ (black squares) and C57Bl/6 (open circles) mice following primary (dotted line) and challenge (solid line) infection. **B.** Numbers of eosinophils recruited to BALF (mean ± S.E.) in EPO^−/−^ and C57Bl/6 mice given primary or challenge Mf infections. **C.** Total serum IgE in µg/ml (mean ± S.E) as measured by ELISA on day 20 post live Mf infection during infection of C57Bl/6, EPO^−/−^ and MBP^−/−^ mice (Mean ± S.E.). **D.** EPO contributes to lung hyper-responsiveness following primary, but not challenge infection. Penh values were measured in conscious, unrestrained mice administered with increasing doses of the aerosolized bronchoconstrictor, methacholine. Mean Penh ± S.E. measurement at day 5, 10 and 20 post live Mf challenge. Graphs show mean Penh ± S.E. **E.** The number of breaths per minute of naïve and infected EPO^−/−^ and WT mice This figure represents data from two independent experiments with 4–6 individual mice per group. *represents a significant difference at p<0.05, ** p<0.01 ****p<0.0001 between groups of gene-targeted mice and C57Bl/6 mice given the same infection regimen.

In order to investigate whether the protective role of eosinophils seen in primary Mf infection and indeed the lung pathology associated with Mf infection is mediated by eosinophil granule proteins, we infected mice specifically lacking either eosinophil peroxidase (EPO) or major basic protein (MBP) and investigated their ability to clear Mf and their development of nematode-induced pathology. EPO^−/−^ and wild-type mice were infected with *B. malayi* Mf alone (primary infection) or following immunisation with Mf antigen (challenge infection). EPO was not necessary for clearance of Mf in either a primary or a challenge infection (Figure 4A). Thus, although eosinophils are required for clearance of primary Mf infection, the mechanism of killing is not dependent upon degranulation of EPO.

The absence of EPO did not significantly alter the recruitment of total leucocytes or non-eosinophilic cells into the lung. Eosinophil infiltration into the lungs was not altered during primary infection in EPO^−/−^ mice, however during challenge infection EPO^−/−^ mice had a reduced eosinophilia in comparison to WT mice at d10 p.i. (p<0.0001) (Figure 4B). No mast cells or basophils were observed in the lungs of either WT or EPO^−/−^ mice. EPO may have an autocrine effect on eosinophil recruitment.

Antigen-specific immunoglobulin isotypes were measured in EPO^−/−^ and WT mice infected with *B. malayi* Mf 20 days pi. As previously shown by our group following Mf infection [23], [24], high levels of antigen-specific IgG1, IgG2a, IgG2c, IgG3 and IgM were found. However there were no significant differences between the levels of these isotypes between infected EPO^−/−^ and wild-type mice (data not shown). Interestingly, there was a significantly higher level of IgE in challenge-infected EPO^−/−^ (but not MBP-1^−/−^) mice in comparison to wild-type mice (p<0.05) suggesting that EPO directly/indirectly regulates IgE and may account for the high IgE in eosinophil-less mice (Figure 4C). Despite these differences in IgE levels between EPO^−/−^ and WT mice, neither IL-4 not the other splenocyte cytokine responses tested (IL-5, IL-13, IL-10 and IFN-γ) differed significantly between the groups of mice (data not shown).

In order to investigate whether EPO is involved in nematode-induced lung dysfunction during primary or challenge infection, lung function was measured by whole body plethysmography at 5, 10 and 20 days following challenge. WT mice displayed physiological changes in respiratory function during primary infection however, EPO^−/−^ mice appeared to be protected from *B. malayi*-induced changes at each time point examined (Figure 4D). There were no differences in Penh between EPO^−/−^ and WT mice during challenge Mf infection. Interestingly absence of EPO also appeared to alter the breathing rate of mice during primary infection (Figure 4E). No differences were observed in goblet cell number or in the level of deposition of collagen or fibrin in infected lungs in the absence of EPO (data not shown).

### Major basic protein-1 is not involved in respiratory changes or Mf clearance in primary infection

In order to investigate whether MBP-1 is necessary as an effector molecule in the clearance of *B. malayi* Mf and/or the molecule responsible for pathology during filarial infection, groups of MBP-1^−/−^ and WT mice were infected with *B. malayi* Mf. Mf survival and filarial-related pathology was assessed at days 5, 10 and 20 post infection.

**Figure 5:**
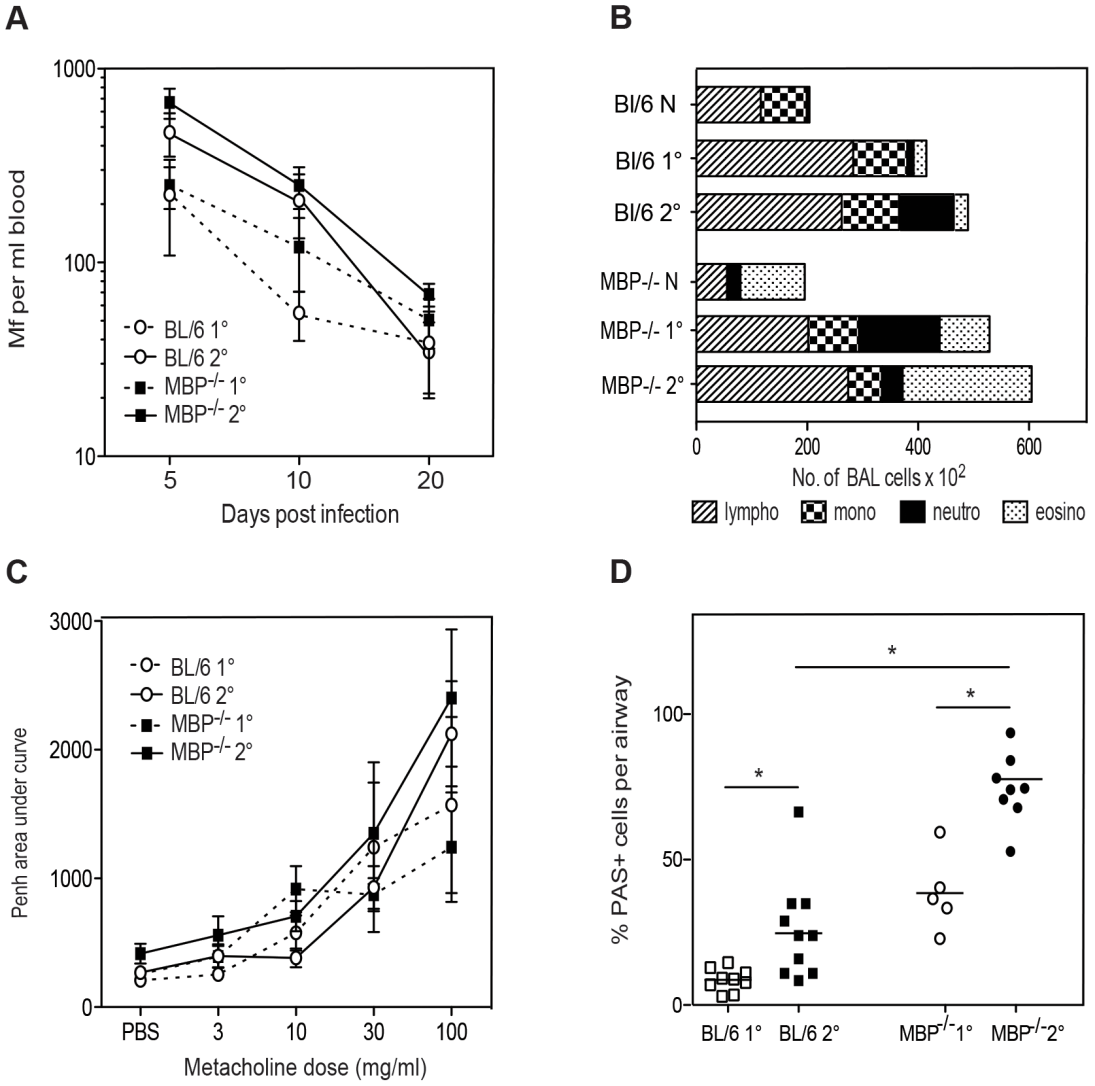
MBP-1 is not required for Mf survival but contributes to pulmonary eosinophil recruitment and goblet cell mucus production following Mf infection. **A.** Mf survival in MBP-1^−/−^ (black squares) and C57Bl/6 (open circles) mice following primary (dotted line) and challenge (solid line) infection. **B.** Mean total and differential cell recruitment to BALF in MBP-1^−/−^ and C57Bl/6 mice given primary or challenge Mf infections. **C.** Lung function (Penh) on day 12 post live Mf challenge showing mean ± S.E. Penh. **D.** Mucus-secreting goblet cells in 6 µm sections of lung, stained with Periodic Acid Schiff (magnification ×40). The graph shows mean ± S.E. percentage of positively staining cells per airway at day 20 post live Mf infection. **A–D.** These graphs represent data from two independent experiments with 4 individual mice per group. *represents a significant difference at p<0.05, ** p<0.01 between groups of gene-targeted mice and C57Bl/6 mice given the same infection regimen.

The absence of MBP-1 did not significantly alter Mf survival during primary infection (Figure 5A). However some parameters of pathology were actually increased in the absence of MBP-1. For example, total numbers of cells recruited into BALF were not significantly different between MBP-1^−/−^ and WT mice infected with Mf, but MBP-1^−/−^ mice had significantly more eosinophils in primary and challenge infection (p<0.05) (Figure 5B). This suggests that MBP-1 release may regulate the recruitment of eosinophils. The filarial antigen-specific antibodies IgG, IgM (data not shown) and total IgE responses did not differ between WT and MBP-1−/− mice (Figure 4C) suggesting that isotype-regulating cytokine responses did not differ in the absence of MBP-1. Furthermore, in accord with this no significant differences were seen between cytokine responses (IL-4, IL-5, IL-13, IL-10 and IFN-γ) from splenocyte restimulation or from BALF in MBP-1^−/−^ and WT mice (data not shown). In addition, while the absence of MBP-1 had little effect upon nematode-induced airway responses (Figure 5C), goblet cells were greatly increased in number in the absence of MBP-1 suggesting that this eosinophil granule protein may modulate goblet cell hyperplasia (Figure 5D). The level of collagen and fibrin deposition in infected lungs did not appear to differ between infected MBP-1^−/−^ and WT mice (data not shown).

## Discussion

In this study we have investigated the dual role of eosinophils as effector cells in parasite clearance and as mediators of pathology. During helminth infection, eosinophils have been widely reported to be involved in protection and they are also linked with helminth and allergy-associated pathology. Indeed, during filarial infection eosinophils have been reported in both of these roles [5], [17], [25], [26]. Eosinophil-mediated killing of filarial nematodes in particular has been the subject of much attention [3], [5], [6], [8], [13], [14], [16], [17], [26]. Interestingly within the filarial nematodes, the efficacy of eosinophils as effector cells varies with both the host and nematode species concerned. In the present study, we have examined the role of eosinophils during infection with the human parasite and causative agent of human lymphatic filariasis, *B. malayi*. Using mice that specifically lack eosinophils [1] or the eosinophil granule proteins, EPO [27] and MBP-1 [28], we definitively confirm and extend our previous results that eosinophils are needed for protection against primary, but not challenge infection of *B. malayi* Mf [5]. We also show that this protection is not solely dependent upon release of either EPO or MBP-1. Additionally, we show that eosinophils contribute to nematode-induced lung pathology and impairment of lung function, and EPO and MBP-1 alone can regulate nematode-induced IgE responses and goblet cell mucus production respectively (results are summarised in Table S1).

In accord with our Mf results, eosinophils are also necessary for killing primary, but not secondary, L3 infections of *B. malayi* [26]. Several studies have shown that eosinophilic granules can be directly toxic to *Brugia* sp. *in vitro*. For example, MBP-1, EPO, eosinophil cationic protein (ECP), and eosinophil-derived neurotoxin (EDN) can all kill Mf, and EPO is particularly potent in this regard [13]. However, our present *in vivo* studies showed that absence of EPO or MBP-1 alone is not sufficient to abrogate Mf clearance. Similarly, survival of *B. pahangi* L3 ip was not altered in either EPO^−/−^ or MBP-1^−/−^ mice [26]. Our studies used *B. malayi* microfilariae alone in the absence of adult worm infection, which lead primarily to induction of type 1 responses, while secondary and challenge Mf infections are known to induce strong type 2 responses as is more usual in natural filarial infection [23], [24], [29]. To date, however, the literature using different *Brugia* sp. nematode stages is remarkably consistent and suggests that eosinophilic granules are either redundant in their ability to kill Mf and L3 of *Brugia* sp. or that eosinophil-dependent clearance of parasites during primary infection is independent of granule deposition and is a downstream function of eosinophil presence such as regulation of responses via eosinophil cytokine release.

Studies using a rodent filarial nematode, *Litosomoides sigmodontis*, show differing results. While survival of a primary infection of *L. sigmodontis* is not altered in eosinophil-deficient mice; eosinophil-intact mice have enhanced nematode development, the established nematodes are significantly longer and the infections achieve patency more rapidly suggesting that eosinophil presence drives faster nematode development [16]. Another study showed that absence of either EPO or MBP-1 resulted in greater adult *L. sigmodontis* establishment following L3 infection, and longer female nematodes, although worms did not survive until patency [14]. This latter study suggests a role for eosinophils in protection against this rodent nematode, although their role may be indirect. Indeed, the absence of eosinophil granule proteins caused several downstream alterations in cytokine responses [14]. For example, thoracic cavity macrophages produced higher IL-10 in both infected MBP-1^−/−^ and EPO^−/−^ mice, while thoracic cavity and splenic T cells had reduced IL-4 production in both strains and reduced IL-5 production in EPO^−/−^ mice [14]. Thus, there is a precedent in this rodent-filarial nematode model for eosinophil granule proteins exerting a role in protection indirectly by cytokine production, eosinophil-induction or activation of other effector cells [14].

Protective immunity against a related filarial worm, *Strongyloides stercoralis*, shows redundancy in the mechanisms mediated by neutrophils and eosinophils. In the primary immune response, larvae are killed either via an eosinophil MBP-1-dependent mechanism, or by a neutrophil myeloperoxidase (MPO)-dependent mechanism. Neutrophil MPO, but not eosinophils, are required for challenge protective immunity [30]. The importance of eosinophils was shown in antibody-treated WT mice while PHIL mice had developed a compensatory protective mechanism. The observation in our study that mast cells are significantly up-regulated in PHIL mice could provide one such compensatory mechanism. Redundancy of granulocyte killing mechanisms has yet to be investigated in models using human filarial nematodes, however, unlike *S. stercoralis*, we have previously shown that neutrophils are not necessary for killing a primary or challenge *B. malayi* Mf infection [31].

In both PHIL and EPO^−/−^ mice, IgE levels were significantly higher than WT mice. This suggests that mouse eosinophils down-regulate induction of Mf-induced IgE. However the mechanism for this is not clear as mouse eosinophils lack FcR for IgE, including CD23, the low affinity IgE receptor that negatively regulates IgE production. Filarial nematode-specific IgG and IgM responses in both PHIL and eosinophil granule-less mice were similar to those found in our previous work [23], [24]. As IgE regulation was also dependent on EPO release, it is possible that alterations in the cytokine milieu due to the absence of eosinophils or their granules may have led to dysregulation of IgE levels or indeed that EPO itself directly regulates IgE [14].

Eosinophils themselves are known to produce a variety of cytokines, including IL-4, IL-3, IL-6, IL-13, IL-10, IL-25, GM-CSF, TGFβ1 and TNFα [32], [33]. Furthermore, eosinophils have been shown in several models to be important for recruitment and activation of effector Th2 cells. They are thought to drive type 2 responses principally by producing IL-4 and IL-13 very early in the immune response, presenting antigen to naïve CD4+ T cells and by secreting chemoattractants that further recruit Th2 effector cells. Indeed the absence of eosinophils in several mouse models of acute and chronic allergic inflammation is accompanied by attenuated Th2 immunity [32], [33]. However, in our experiments with Mf infection we did not find significant changes in Type 2 cytokine responses. The IFN-γ was sustained in primary infection in the absence of eosinophils, which could suggest a rise in type 1 responses or it could reflect the fact that Mf, which induce IFN-γ, survive for longer in these mice. While we can not rule out the possibility that the sampling time-points for type 2 cytokines were sub-optimal, our measures of other Type 2-mediated parameters such as IgE production and goblet cell metaplasia, which reflect historical type 2 cytokine production, were actually enhanced in eosinophil absence. In addition downstream components of type 2 immunity, such as IgE and goblet cell metaplasia, were increased in the absence of eosinophils. Overall, our results suggest that the type 2 responses generated during Mf challenge infection are not entirely dependent upon eosinophil presence and could be compensated for by non-eosinophil cell sources such as mast cells, NK T cells, γδ T cells or basophils [24]. Indeed an increase in mast cells was seen in PHIL mice following challenge infection. Future work will dissect the need for eosinophil-derived IL-4 or IL-13 in differentiation and mobilisation of Th2 cells in different pathogen models.

The implication from our previous work using the *Brugia*-Mf mouse model is that eosinophils while acting as effector cells in primary infection against Mf, may in fact mediate pathological damage during challenge and/or chronic infection [5]. In wild type mice we have shown significant eosinophil recruitment to the lung occurs during primary and challenge infection. The absence of MBP-1 alone in the above experiments increased eosinophil levels in naïve and infected mice, while eosinophil number was reduced in EPO^−/−^ mice following challenge infection. However, evidence for the role of eosinophils and/or their granules in recruitment of cells to the site of infection/damage varies. For example, recruitment of cells into subcutaneous diffusion chambers following primary *S. stercoralis* infection of WT, EPO^−/−^ and MBP-1^−/−^ mice did not differ [30], eosinophil recruitment to the peritoneal cavity following *B. pahangi* L3 infection of EPO^−/−^ but not MBP-1^−/−^ mice was reduced [26] while eosinophil recruitment to the thoracic cavity of *L. sigmodontis* infected EPO^−/−^ mice increased [14]. Currently the precise role of eosinophil granule proteins in haemopoiesis, and/or their autocrine effects on bone marrow progenitors, differentiation of lineage-committed precursors, and the survival of mature metamyelocytes in circulation is the subject of investigation. Indeed, recent work has shown that mice lacking both MBP-1 and EPO have very few eosinophils and appear to regulate either eosinophil precursor haemopoiesis, survival and/or development by a mechanism targeting eosinophil progenitor survival [34].

Our study showed that the absence of eosinophils ameliorated nematode challenge-induced alterations in lung physiology. However, this was not explained by changes in collagen deposition and mucus production was greater in PHIL mice. In EPO^−/−^ mice there was also a reduction in nematode-infection induced lung hyper-responsiveness and in the baseline respiratory rate, suggesting that release of EPO plays a role in nematode-induced lung pathology. However, although MBP-1 is deposited on lung epithelial tissue following challenge with live *B. malayi* Mf, our results suggested that eosinophilic release of MBP-1 is not involved in Mf-induced alterations in lung physiology [17]. Interestingly, the absence of MBP-1 resulted in increased mucus-producing goblet cells suggesting that MBP-1 itself, and not EPO, may affect goblet cell metaplasia. We conclude that goblet cell metaplasia is not involved in nematode challenge-induced respiratory changes. Further studies will be needed to pinpoint the exact cause of eosinophil-mediated nematode-induced changes in lung function. In an OVA-challenge mouse model of asthma, absence of neither EPO nor MBP-1 alters AHR [27], [28]. Indeed, in the pathological condition in humans associated with filarial infection, tropical pulmonary eosinophilia (TPE), levels of pathology appear to be most closely correlated with presence of the eosinophil granule protein, eosinophil-derived neurotoxin (EDN) [25]. This merits further investigation, however, currently mice deficient in this granule protein are not available.

Studies of pulmonary cell recruitment in allergy models have shown a very different picture to that of nematode-induced pathology. PHIL mice used in an OVA-challenge allergy model, do not develop PAS^+^ lung goblet cells, unless they are adoptively transferred with T cells and eosinophils suggesting that eosinophils are needed for the recruitment of Th2 cells to the lung [1], [35]. In a different eosinophil-less mouse, ΔDbl-GATA, OVA-challenge suggested that eosinophils are not involved in inflammation or AHR but are involved in lung remodelling responses such as collagen deposition and increases in airway smooth muscle [2]. Challenging a number of eosinophil-defective mice with *Aspergillus* allergen, Fulkerson *et al*. (2006) also reported that eosinophil absence is associated with a significant reduction in pulmonary Th2 gene expression and mucus production [36]. However, in a *Nippostrongylus brasiliensis* nematode-mouse model of lung inflammation, eosinophils are not required for pulmonary T cell recruitment, IgE production or worm expulsion during primary infection while they do play a limited role in activation or recruitment of CD4^+^ T cells to the lung following challenge [37]. Thus, data from these studies highlight the complexity of pulmonary immune responses and also highlight differences between the role of eosinophils in allergy and different nematode-induced pathology models.

Overall our results reveal that eosinophils actively participate in protective immune responses against a nematode parasite. In addition, eosinophils and their granules are influential as negative regulators of specific parasite-induced lung inflammatory and adaptive responses. This study highlights the importance of eosinophils as effector cells, as damage-inducing cells and as supervisory cells that shape both innate and adaptive immunity.

## Materials and Methods

### Ethics statement

Animal experiments were conducted in accordance with our project licence (PPL 70/7243), which was approved by the Home Office under the Animal Scientific Procedures Act (1986). The project was approved by the local Ethical Review Committee at the Royal Veterinary College.

### Mice

PHIL mice (deficient in eosinophils) [1], MBP-1^−/−^ mice [28] and EPO^−/−^ mice [27] on a C57Bl/6 background were obtained from Prof JJ Lee (Mayo Clinic, Arizona) and bred at the Royal Veterinary College. PHIL mice were generated using a cytocidal protein under the control of the EPO promoter, thus ablating EPO-expressing cells [1]. Wild type C57Bl/6 mice were purchased from Harlan UK at 6–8 weeks of age. All mice used in experiments were male, age-matched between groups and housed in individually-ventilated cages.

### Parasites

*B. malayi*-infected gerbils (*Meriones unguiculatus*) were purchased from TRS Labs (Georgia, USA) and were housed in standard conditions. Mf were obtained by peritoneal lavage with RPMI 1640 and gerbil cells were removed by centrifugation over lymphocyte separation media (Flow Labs, McLean, VA, USA) [38]. Soluble Mf extract was prepared as described previously [39].

### Infection protocol

Groups of four to six individual, eight week old, male C57Bl/6 wild-type (WT) mice and/or gene-targeted mice were either left uninfected (naïve), or they were injected with 2×10^5^ *B. malayi* Mf i.v. (primary (1°) infection) or they were immunised on three occasions with 200 µg soluble Mf extract s.c. prior to challenge with 200,000 *B. malayi* Mf i.v. (challenge (2°) infection) (Figure 1). At days 5–6, 10–12 and 20–21 post infection with Mf, immunological and pathological parameters of mice were measured in all groups of mice. Parasitaemia was monitored as previously described [39].

### Whole body plethysmography

Whole body plethysmography was used to determine enhanced pause (Penh). While there is controversy over the validity of Penh as a parameter for lung mechanics or resistance [40], [41], [42], [43], we use our measurements as an added indicator of physiological change during the infection process in conjunction with a number of other cellular histopathological measurements in the lung. A whole body plethysmograph (EMMS, Bordon, Hants) was used according to the manufacturer's instructions to determine Penh and breathing frequency in mice. Briefly, mice were placed in plethysmograph chambers and allowed to acclimatise for 20 min before a 4 min control period was recorded. Mice were exposed to varying concentrations of methacholine, up to a maximum of 100 mg/ml, and lung function was monitored for 5 min following each methacholine challenge. Mice were rested for 5 min between each challenge to allow lung function to return to baseline before the next challenge.

### Bronchoalveolar lavage

A cannula was inserted into the trachea, and bronchoalveolar lavage was performed with 900 µl PBS. BALF was centrifuged at 13,000× g for 10 min, supernatant was removed and stored at −20°C. Cells were re-suspended, counted and cyto-centrifuged onto a microscope slide at 800× g for 5 min. Slides were air dried for 20 min, prior to fixation for 2 min in 50% methanol and 50% acetone. Fixed slides were stained with May-Gruenwald and Giemsa (10% solution in Giemsa buffer).

### Lung histology

Lungs were fixed in neutral buffered formalin, dehydrated in increasing concentrations of ethanol and processed in a Tissue Tek processor. Tissues were embedded in wax and 6 µm sections were cut. Sections were stained with haematoxylin and eosin, Periodic Acid Schiff or Martius Scarlet Blue.

### Measurement of Mf-specific immunoglobulin isotypes

Mf-specific immunoglobulin levels were measured by ELISA [5]. Briefly 96-well plates were coated overnight at 4°C with 1 µg/ml soluble Mf extract (MfAg) in 50 µl carbonate buffer (pH 9.6). After blocking each well with 10% FCS in carbonate buffer, the plates were incubated with individual mouse sera diluted 1∶50 in PBS 0.5% Tween-20. Antigen-specific antibodies were detected using horseradish peroxidase (HRP)-conjugated goat anti-IgM (Southern Biotechnology Associates, Birmingham, AL, USA; SBA 1020-05), anti-IgG1 (SBA1070-05), anti-IgG2a (SBA 1080-05), anti-IgG2b (SBA 1090-05) or anti-IgG3 (SBA 1100-05). 3,3′,5,5′-Tetramethylbenzidine (TMB) (Sigma) was used as the substrate. Plates were read at 405 nm. Total IgE was measured by ELISA as previously described [44].

### Cell culture and cytokine ELISA

Spleen cells were cultured at 5×10^6^ cells/ml in RPMI plus 5% FCS and 10 µg/ml MfAg, 5 µg/ml concanavalin A (Con A) or 1 µg/ml anti-CD3 as previously described [45]. Cells were incubated for 72 h at 37°C and supernatants were removed for cytokine analysis. The concentration of the cytokines, IL-4, IL-5, IL-10 and IFN-γ in the recovered supernatants was determined by sandwich ELISA. Purified and biotinylated monoclonal antibody pairs, 11B11 and BVD6-24G2 (IL-4), TRFK5 and TRFK4 (IL-5), JES5-2A5 and SXC-1 (IL-10), R46A2 and XMG1.2 (IFN-γ) were purchased from BD Pharmingen (San Diego, CA, USA). Cytokine concentrations were measured against recombinant murine cytokine standards as previously described [45]. Briefly, each well was coated with capture antibody overnight at 4°C. Plates were washed and incubated with supernatant or recombinant cytokine standard for 2 h at 37°C. Following washing, biotinylated anti-mouse cytokine antibodies (2 µg/ml) were incubated for 45 min at 37°C, wells were washed again and incubated with streptavidin-HRP (R&D) for 30 min at 37°C. In the IL-10 ELISA, extravidin-alkaline phosphatase (AP) (Sigma) was used at 1 µg/ml. Finally, plates were washed and developed either with TMB substrate for HRP-conjugated antibodies (BD Pharmingen) or for AP-conjugated antibody, p-nitrophenyl phosphate (pNPP) was used as a substrate. Plates were read at 450 nm for TMB and 405 nm for pNPP. IL-13 was measured using a Quantikine ELISA kit according to the manufacturer's instructions (R&D Biosciences).

### Statistical analysis

All data are expressed as mean ± SE. One-way ANOVA with Bonferroni's post-hoc analysis was used for intergroup comparisons between infected PHIL, MBP-1^−/−^, EPO^−/−^ and WT mice. *P*-values lower than 0.05 were considered statistically significant. Prism (Graphpad Software Inc.) statistical analysis software was used to determine significance.

## Supporting Information

Figure S1 Collagen deposition in the lungs of naïve, primary and challenge infected WT and PHIL mice day 12 post live Mf challenge infection. Lungs were cut into 6 µm lung sections and stained with Martius Scarlet Blue. Magnification ×20. This figure shows data from day 12 post live Mf challenge and represents data from two independent experiments with 6 individual mice per group.
(TIF)

Table S1 Summary of Mf survival, immune responses and parameters of pathology. Results are summarised from PHIL, EPO^−/−^ and MBP-1^−/−^ mice in comparison to C57Bl/6 mice (WT) given either a primary live infection of *B. malayi* Mf (1°) or a live Mf challenge infection post immunisation (2°). ↑ represents significantly increased responses compared to C57Bl/6 mice given the same infection regimen, while, ↓ represents a significantly decreased response compared to C57Bl/6 mice given the same infection regimen. ns represents parameters in which there is no significant difference from C57Bl/6 mice given the same infection regimen. WBP stands for whole body plethysmography.
(DOC)
