# Supplementary figures and images for: Eosinophils Are Important for Protection, Immunoregulation and Pathology during Infection with Nematode Microfilariae
Source: PLoS Pathog. 2014 Mar 13;10(3):e1003988. doi: 10.1371/journal.ppat.1003988 (PMC3953434; doi:10.1371/journal.ppat.1003988)

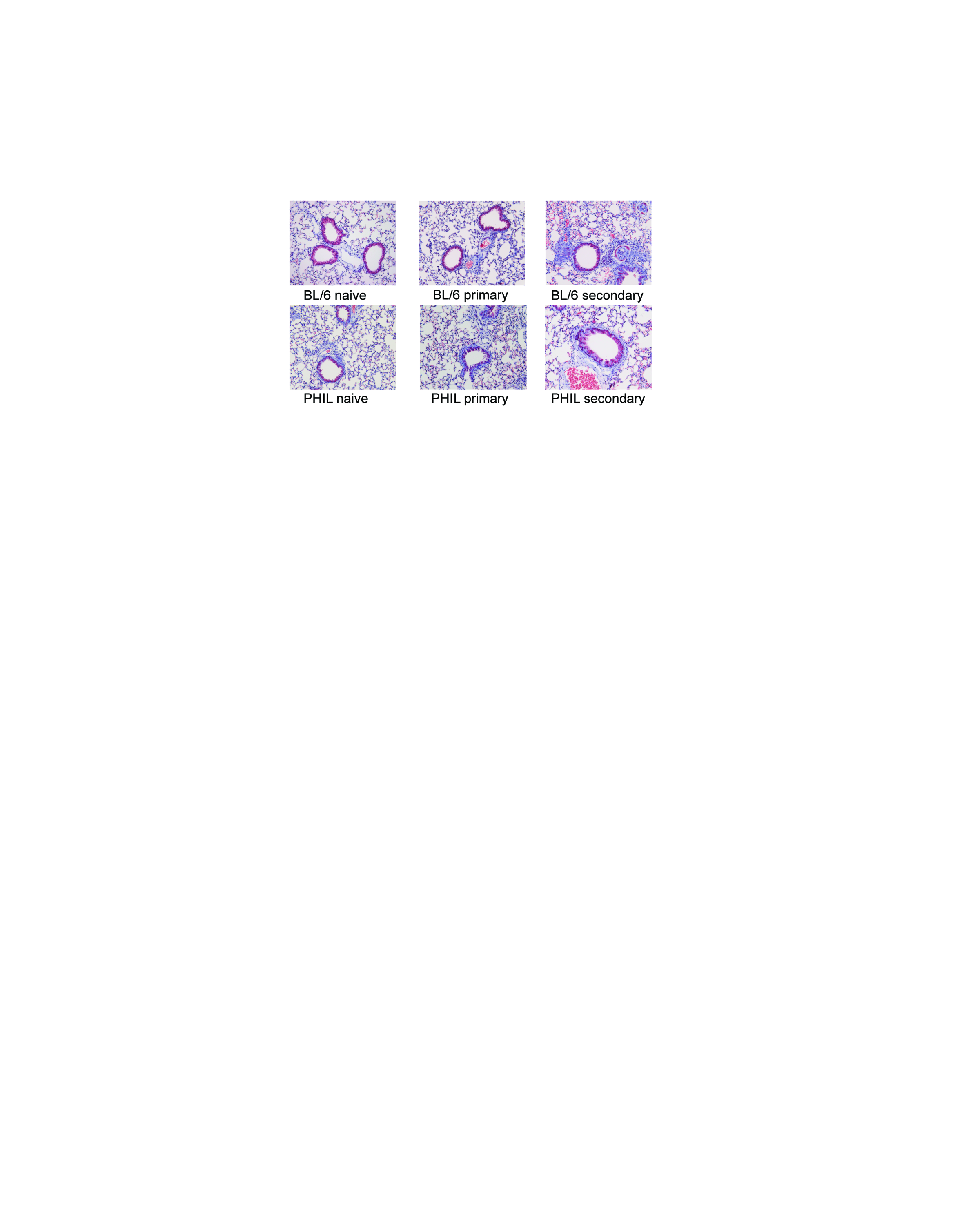

Supplement: Figure S1 — Collagen deposition in the lungs of naïve, primary and challenge infected WT and PHIL mice day 12 post live Mf challenge infection. Lungs were cut into 6 µm lung sections and stained with Martius Scarlet Blue. Magnification ×20. This figure shows data from day 12 post live Mf challenge and represents data from two independent experiments with 6 individual mice per group. (TIF) [file ppat.1003988.s001.tif]
