# Supplementary material for: Eosinophils Are Important for Protection, Immunoregulation and Pathology during Infection with Nematode Microfilariae
Source: PLoS Pathog. 2014 Mar 13;10(3):e1003988. doi: 10.1371/journal.ppat.1003988 (PMC3953434; doi:10.1371/journal.ppat.1003988)
Supplement: Table S1 — Summary of Mf survival, immune responses and parameters of pathology. Results are summarised from PHIL, EPO−/− and MBP-1−/− mice in comparison to C57Bl/6 mice (WT) given either a primary live infection of B. malayi Mf (1°) or a live Mf challenge infection post immunisation (2°). ↑ represents significantly increased responses compared to C57Bl/6 mice given the same infection regimen, while, ↓ represents a significantly decreased response compared to C57Bl/6 mice given the same infection regimen. ns represents parameters in which there is no significant difference from C57Bl/6 mice given the same infection regimen. WBP stands for whole body plethysmography. (DOC) [file ppat.1003988.s002.doc]

Table S1. Summary of Mf survival, immune responses and parameters of pathology.

|  | **PHIL** | | **EPO-/-** | | **MBP-/-** | |
| --- | --- | --- | --- | --- | --- | --- |
|  | **1°** | **2°** | **1°** | **2°** | **1°** | **2°** |
| **Mf Survival** | ↑ | ns | ns | ns | ns | ns |
| **Eosinophil** | ↓ | ↓ | ns | ↓ | ↑ | ↑ |
| **Mast cell** | ns | ↑ | - | - | - | - |
| **Ab** | ns | ↑ IgE | ns | ↑ IgE | ns | ns |
| **Cytokines** | ↑ IFNγ | ns | ns | ns | ns | ns |
| **WBP** | ↓ (ns) | ↓ | ↓ | ns | ns | ns |
| **Lung Goblet Cell** | ns | ↑ | ns | ns | ↑ (ns) | ↑ |
